# Supplementary material for: Immunotherapy and Advanced Vulvar Cancer: A Systematic Review and Meta-Analysis of Survival and Safety Outcomes
Source: Cancers (Basel). 2025 Jul 19;17(14):2392. doi: 10.3390/cancers17142392 (PMC12294087; doi:10.3390/cancers17142392)
Supplement: Supplementary file 1 [file cancers-17-02392-s001.zip › Supplementary Material S1.pdf]

Systematic Review Search Strategy: Immunotherapy in Advanced Vulvar Cancer

Databases Searched:

- PubMed/MEDLINE
- Embase
- Scopus
- Cochrane Library

Search Date Range:
From database inception to May 2025

Search Terms and Strategy (example for PubMed):

(vulvar cancer OR vulvar neoplasms OR vulvar squamous cell carcinoma OR VSCC) AND

(immunotherapy OR immune checkpoint inhibitors OR checkpoint blockade OR PD-1 OR PD-L1 OR CTLA-4 OR pembrolizumab OR nivolumab OR ipilimumab OR cemiplimab)

MeSH Terms and Free Text (PubMed example):

- "Vulvar Neoplasms"[MeSH]
- "Immunotherapy"[MeSH]
- "Programmed Cell Death 1 Receptor"[MeSH]
- "Programmed Cell Death 1 Ligand 1 Protein"[MeSH]
- "Cytotoxic T-Lymphocyte Antigen 4"[MeSH]
- Free-text keywords: vulvar cancer, VSCC, checkpoint inhibitors, PD-1, PD-L1, CTLA-4, pembrolizumab, nivolumab, cemiplimab, ipilimumab

Boolean Operators: AND/OR used to refine and expand results appropriately.

Limits Applied:

- Language: English
- Human subjects
- Study types: Clinical trials, observational studies, cohort studies, case series, and case reports with clinical data

Additional Search Methods:

- Manual reference screening of included studies and relevant reviews
- Grey literature (e.g., conference abstracts only when full data are extractable)

Search Record Management:
All records were imported and de-duplicated using EndNote and Rayyan. Screening was performed by two independent reviewers.
