# Supplementary material for: Immunotherapy and Advanced Vulvar Cancer: A Systematic Review and Meta-Analysis of Survival and Safety Outcomes
Source: Cancers (Basel). 2025 Jul 19;17(14):2392. doi: 10.3390/cancers17142392 (PMC12294087; doi:10.3390/cancers17142392)
Supplement: Supplementary file 1 [file cancers-17-02392-s001.zip › Table S1.pdf]

Table S1. PICO framework.

**Population (P)**	Patients diagnosed with advanced (unresectable, recurrent, or metastatic) VSCC, with or without prior systemic and/or local therapy, immunotherapy-naive.
**Intervention (I)**	ICIs, including anti-PD-1 (pembrolizumab, nivolumab), anti-CTLA-4 (ipilimumab), and anti-PD-L1 (cemiplimab), as monotherapy or in combination.
**Comparison (C)**	When comparator arm available: standard chemotherapy or best supportive care.
**Outcomes (O)**	Primary Outcomes: ORR, PFS, and OS. Secondary Outcomes: Safety, incidence of treatment-related AEs (Grade 1–5), and biomarker correlations (PD-L1 and HPV status).
**Studies (S)**	Full text RCTs, Phase I-III trials written in English, published in peer-reviewed journals.

AE, adverse event; CTLA-4, Cytotoxic T-Lymphocyte Antigen 4; HPV, human papillomavirus; ICI, immune checkpoint inhibitors; MSI, microsatellite instability; ORR, overall response rate; OS, overall survival; PD-L1, programmed death ligand-1; PFS, progression-free survival; RCT(s), randomized controlled trial(s); VSCC, vulvar squamous cell carcinoma.
