# Supplementary material for: Immunotherapy and Advanced Vulvar Cancer: A Systematic Review and Meta-Analysis of Survival and Safety Outcomes
Source: Cancers (Basel). 2025 Jul 19;17(14):2392. doi: 10.3390/cancers17142392 (PMC12294087; doi:10.3390/cancers17142392)
Supplement: Supplementary file 1 [file cancers-17-02392-s001.zip › Table S2.pdf]

Table S2. MINORS tool's results. Each item was scored from 0 (not reported) to 2 (reported and adequate).

Study	Clearly stated aim	Consecutive patients	Prospective data collection	Appropriate endpoints	Unbiased endpoint assessment	Adequate follow-up	Loss to follow-up <5%	Sample size calculation	Total
KEYNOTE-158 [37]	2	1	2	2	1	2	2	0	12
KEYNOTE-028 [24]	2	1	2	2	1	2	2	0	12
CheckMate 358 [25]	2	2	2	2	2	2	2	0	14
PEVOsq [38]	2	2	2	1	1	2	2	0	13
SWOG S1609 [40]	2	2	2	2	1	1	1	0	11
Yeku et al. [39]	2	1	2	1	1	1	1	0	10
