# Supplementary material for: Immunotherapy and Advanced Vulvar Cancer: A Systematic Review and Meta-Analysis of Survival and Safety Outcomes
Source: Cancers (Basel). 2025 Jul 19;17(14):2392. doi: 10.3390/cancers17142392 (PMC12294087; doi:10.3390/cancers17142392)
Supplement: Supplementary file 1 [file cancers-17-02392-s001.zip › Table S3.pdf]

Table S3. GRADE study assessment.

Outcome	No. of studies	Study design	Risk of bias	Inconsistency	Indirectness	Imprecision	Publication bias	Overall
ORR	6	Non-RCT	Moderate	Moderate	No	Serious	Possible	●●○○ Low
PFS	4	Non-RCT	Moderate	Serious	No	Serious	NA	●●○○ Low
OS	3	Non-RCT	Moderate	Serious	No	Serious	NA	●●○○ Low
Safety	6	Non-RCT	Moderate	Low	No	Moderate	NA	●●●○ Moderate

NA, not available; PFS, progression-free survival; ORR, overall response rate; OS, overall survival; RCT, randomized controlled trial
