# Supplementary figures and images for: Immunotherapy and Advanced Vulvar Cancer: A Systematic Review and Meta-Analysis of Survival and Safety Outcomes
Source: Cancers (Basel). 2025 Jul 19;17(14):2392. doi: 10.3390/cancers17142392 (PMC12294087; doi:10.3390/cancers17142392)

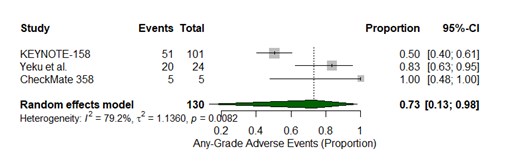

Supplement: Supplementary file 1 [file cancers-17-02392-s001.zip › Figure S1.png]

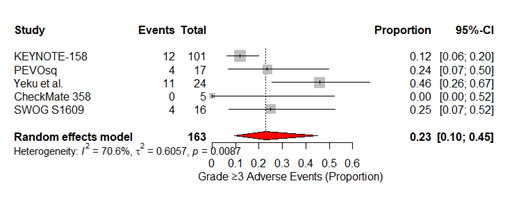

Supplement: Supplementary file 1 [file cancers-17-02392-s001.zip › Figure S2.png]

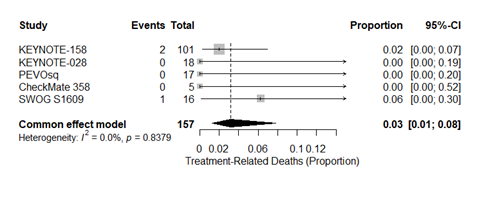

Supplement: Supplementary file 1 [file cancers-17-02392-s001.zip › Figure S3.png]
